# Supplementary material for: The Evaluation of Vascular Endothelial Growth Factor A (VEGFA) and VEGFR2 Receptor as Prognostic Biomarkers in Bladder Cancer
Source: Diagnostics (Basel). 2023 Apr 19;13(8):1471. doi: 10.3390/diagnostics13081471 (PMC10137622; doi:10.3390/diagnostics13081471)
Supplement: Supplementary file 1 [file diagnostics-13-01471-s001.zip › diagnostics-2279890-supplementary.pdf]

diagnostics

Supplementary Materials: The Evaluation of Vascular Endothelial Growth Factor A (VEGFA) and VEGFR2 Receptor as Prognostic Biomarkers in Bladder Cancer

Meryem El Azzouzi [1,2,†], Hajar El Ahanidi [1,3,†], Chaimae Hafidi Alaoui [1,4], Imane Chaoui [1], Laila Benbacer [1], Mohammed Tetou [2,5], Ilias Hassan [2,5], Mounia Bensaid [6], Mohamed Oukabli [2,6], Ahmed Ameur [2,5], Abderrahmane Al Bouzidi [2], Mohammed Attaleb [1,7,*,‡] and Mohammed El Mzibri [1,‡]

Table S1. Association between VEGFA -460 T/C polymorphism and clinical pathological features of BC patients

	N	Genotype				Allele		
		VEGFA -460 T/C						
		CC (%)	CT (%)	TT (%)	*p*	C (%)	T (%)	*p*
Gender								
Male	68	36 (52.9)	14(20.6)	18(26.5)	0.36	86(63.2)	50(36.8)	0.29
Female	2	0	1(50)	1(50)		1(25)	3(75)	
Age								
≤ 50	1	1(100)	0	0		2(100)	0	
50-70	44	23(52.3)	8(18.2)	13(29.5)	0.51	54(61.4)	34(38.6)	0.39
>70	25	12(48)	7(28)	6(24)		31(62)	19(38)	
Smoking								
Yes	28	18(64.3)	8(28.6)	2(7.1)	0.02	44(78.6)	12(21.4)	0.003
No	42	18(42.9)	7(16.6)	17(40.5)		43(51.2)	41(48.8)	
Stage								
≤ Pt1	52	27(51.9)	9(17.3)	16(30.8)	0.79	63(60.6)	41(39.4)	0.75
> Pt1	18	9(50)	6(33.4)	3(16.6)		24(66.6)	12(33.4)	
Grade								
Low	27	15(55.6)	5(18.5)	7(25.9)	0.51	35(64.8)	19(35.2)	0.41
High	43	21(48.8)	10(23.3)	12(27.9)		52(60.5)	34(39.5)	
Recurrence								
Yes	12	8(66.7)	1(8.3)	3(25)	0.37	17(70.8)	7(29.2)	0.24
No	40	19(47.5)	8(20)	13(32.5)		46(57.5)	34(42.5)	
Progression								
Yes	5	2(40)	2(40)	1(20)	0.89	6(60)	4(40)	0.86
No	47	25(53.2)	7(14.9)	15(31.9)		57(60.6)	37(39.4)	

Table S2. Association between VEGFA -2578 C/A polymorphism and clinical pathological features of BC patients.

	N	Genotype				Allele		
		VEGFA -2578 C/A						
		AA (%)	AC (%)	CC (%)	*p*	A (%)	C (%)	*p*
Gender								
Male	68	21(30.9)	28(41.2)	19(27.9)	0.21	70(51.5)	66(48.5)	0.21
Female	2	1(50)	1(50)	0		3(75)	1(25)	
Age								
≤ 50	1	1(100)	0	0		2 (100)	0	
50-70	44	12(27.3)	22(50)	10(22.7)	0.40	46(52.3)	42(47.7)	0.35
>70	25	9(36)	7(28)	9(36)		25(50)	25(50)	
Smoking								
Yes	28	11(39.3)	13(46.4)	4(14.3)	0.07	35(62.5)	21(37.5)	0.06
No	42	11(26.2)	16(38.1)	15(35.7)		38(45.2)	46(54.8)	
Stage								
≤ Pt1	52	15(28.8)	21(40.4)	16(30.8)	0.73	51(49)	53(51)	0.71
> Pt1	18	7(38.9)	8(44.4)	3(16.7)		22(61.1)	14(38.9)	
Grade								
Low	27	6(22.2)	13(48.1)	8(29.7)	0.31	25(46.3)	29(53.7)	0.27
High	43	16(37.2)	16(37.2)	11(25.6)		48(55.8)	38(44.2)	
Recurrence								
Yes	12	4(33.3)	5(41.7)	3(25)	0.60	13(54.2)	11(45.8)	0.55
No	40	11(27.5)	16(40)	13(32.5)		38(47.5)	42(52.5)	
Progression								
Yes	5	1(20)	3(60)	1(20)	0.88	5(50)	5(50)	0.87
No	47	14(29.8)	18(38.3)	15(31.9)		46(48.9)	48(51.1)	

Table S3. Association between VEGFA -2549I/D polymorphism and clinical pathological features of BC patients.

	N	Genotype				Allele		
		VEGFA -2549 I/D						
		**II (%)**	**ID (%)**	**DD (%)**	***p***	**I (%)**	**D (%)**	***p***
Gender								
Male	68	22(32.4)	24(35.3)	22(32.3)	0.17	68(50)	68(50)	0.15
Female	2	1(50)	1(50)	0		3(75)	1(25)	
Age								
≤ 50	1	1(100)	0	0	0.56	2(100)	0	0.51
50-70	44	12(27.3)	19(43.2)	13(29.5)		43(48.9)	45(51.1)	
>70	25	10(40)	6(24)	9(36)		26(52)	24(48)	
Smoking								
Yes	28	11(39.3)	11(39.3)	6(21.4)	0.17	33(58.9)	23(41.1)	0.11
No	42	12(28.6)	14(33.3)	16(38.1)		38(45.2)	46(54.8)	
Stage								
≤ Pt1	52	15(28.9)	18(34.6)	19(36.5)	0.53	48(46.2)	56(53.8)	0.47
> Pt1	18	8(44.4)	7(38.9)	3(16.7)		23(63.9)	13(36.1)	
Grade								
Low	27	6(22.3)	10(37)	11(40.7)	0.15	22(40.7)	32(59.3)	0.11
High	43	17(39.5)	15(34.9)	11(25.6)		49(57)	37(37)	
Recurrence								
Yes	12	4(33.3)	3(25)	5(41.7)	0.93	11(45.8)	13(54.2)	0.98
No	40	11(27.5)	15(37.5)	14(35)		37(46.3)	43(53.7)	
Progression								
Yes	5	1(20)	3(60)	1(20)	0.83	5(50)	5(50)	0.79
No	47	14(29.8)	15(31.9)	18(38.3)		43(45.7)	51()	
